# Karyotypes of field mice of the genus *Apodemus* (Mammalia: Rodentia) from China

**DOI:** 10.24272/j.issn.2095-8137.2018.054

**Published:** 2018-05-28

**Authors:** Masaharu Motokawa, Yi Wu, Masashi Harada, Yuta Shintaku, Xue-Long Jiang, Yu-Chun Li

**Affiliations:** 1 Kyoto University Museum, Kyoto University, Kyoto 606-8501, Japan; 2 Key Laboratory of Conservation and Application in Biodiversity of South China, School of Life Sciences, Guangzhou University, Guangzhou Guangdong 510006, China; 3 Laboratory Animal Center, Graduate School of Medicine, Osaka City University, Osaka 545-8585, Japan; 4 Wildlife Research Center, Kyoto University, Sakyo, Kyoto 606-8203, Japan; 5 Japan Monkey Centre, Inuyama, Aichi 484-0081, Japan; 6 Kunming Institute of Zoology, Chinese Academy of Sciences, Kunming Yunnan 650223, China; 7 Marine College, Shandong University (Weihai), Weihai Shandong 264209, China

**Keywords:** Karyotype, Chromosome evolution, Speciation, Taxonomy, Field mice

## Abstract

Karyotypes of four Chinese species of field mice of the genus *Apodemus* were examined, including *Apodemus chevrieri* (diploid chromosome number, 2*n*=48, fundamental number of autosomal arms, FNa=56), *A. draco* (2*n*=48, FNa=48), *A. ilex* (2*n*=48, FNa=48), and *A. latronum* (2*n*=48, FNa=48). Karyotypes of *A. chevrieri*, *A. draco*, and *A. ilex* are reported here for the first time, providing useful information for their species taxonomy. Determining the karyotypes of all species of *Apodemus* in Asia, both in this and previous studies, provides a solid overview of the chromosome evolution and species differentiation of the genus in East Asia. In addition to allopatric speciation, chromosome rearrangements likely played an important role in the formation of the four *Apodemus* species groups as well as speciation within each group in East Asia. For example, increased centromeric heterochromatin in *A. latronum* may have contributed to the post-mating reproductive isolation from the *A. draco*-*A. ilex*-*A. semotus* clade.

## INTRODUCTION

Field mice of the genus *Apodemus* are common murid species widely distributed in the Palearctic region through to the northern part of the Oriental region. The genus currently includes 20 species (Musser et al., 1996; Musser & Carlenton, 2005), which have been characterized into three species groups based on morphological characters from detailed literature review (Musser et al., 1996): that is, *Apodemus* Group (*A. agrarius*, *A. chevrieri*, *A. speciosus*, *A. peninsulae*, *A. latronum*, *A. draco*, *A. semotus*, *A. gurkha*), *Sylvaemus* Group (*A. sylvaticus*, *A. flavicollis*, *A. uralensis*, *A. mystacinus*, *A. fulvipectus*, *A. heremonensis*, *A. alpicola*, *A. arianus*, *A. hyrcanicus*, *A. ponticus*, *A. rusiges*, *A. wardi*), and *Argenteus* Group (*A. argenteus*). The *Apodemus* Group and *Argenteus* Group consist of species distributed in East Asia, whereas species within the *Sylvaemus* Group are found in western Palearctic region. The *A. agrarius* species from the *Apodemus* Group is widely distributed in the Palearctic region from East Asia to Europe. Currently, however, there is still considerable taxonomic confusion regarding the species boundaries and identification of East Asian *Apodemus* species (Musser et al., 1996), especially those distributed in China.

Several phylogenetic studies using genetic approaches were conducted to reveal the species relationship and validity of the above-mentioned species groups (Filippucci et al., 2002; Liu et al., 2004; Michaux et al., 2002; Serizawa et al., 2000; Suzuki et al., 2003, 2008). Suzuki et al. (2008) conducted comprehensive phylogenetic analyses based on mitochondrial and nuclear genes from most species of *Apodemus* and confirmed the distinct lineages of the three species groups, except for *A. gurkha*, which showed an independent lineage from the other species within the *Apodemus* Group.

Concerning the evolutionary history of the genus *Apodemus* in East Asia, Suzuki et al. (2008) determined that the three species groups formed around 6 million years ago (Ma), with the *Apodemus* Group splitting into four ancestral species (*A. agrarius*/*A. chevrieri*, *A. draco* (and *A. ilex*)/*A. semotus*/*A. latronum*, *A. peninsulae*, and *A. speciosus*) around 5 Ma, and then splitting into the currently recognized species around 2 Ma. For these speciation events, Suzuki et al. (2008) assumed that allopatric speciation likely played an important role, followed by range expansion and distribution overlap. The original place for speciation event, however, has not been mentioned and unspecified.

Chromosomal divergence is thought to play a role in reproductive isolation (e.g., King, 1993). Examination of karyotypes of species and populations is important to reconstruct allopatric and sympatric speciation events and clarify the historical changes in species distribution. Species differentiation among congeneric species also participates in cytological reproductive isolation (e.g., King, 1993). While the karyotypes of *Apodemus* species have been relatively well studied (e.g., Matsubara et al., 2004), information on species and populations in China is still limited. Clarification of species karyotypes is important for understanding the diversification of a genus. In this study, we examined the karyotypes of *A. chevrieri*, *A. draco*, *A. ilex*, and *A. latronum* based on specimens collected in China to help fill the gap in current knowledge. Even though the newly reported karyotypes were limited to conventional karyotypes, we expect they will be useful for the evaluation of species taxonomy and will provide an overview of chromosomal evolution and species differentiation. We also examined evolutionary history in consideration of the molecular and chromosomal divergences of *Apodemus* in East Asia.

## MATERIALS AND METHODS

A total of 71 specimens from four *Apodemus* species (*A. chevrieri*, *A. draco*, *A. ilex*, and *A. latronum*) in China were examined. Species identification was made by careful examination of cranial characters following Musser et al. (1996), in addition to external characters and measurements. *Apodemus ilex* (mostly distributed in Yunnan, China) is often considered a synonym of *A. draco* (e.g., Musser & Carlenton, 2005); however, molecular phylogeographic data suggest two species (e.g., Liu et al., 2012). In this study, we considered *A. ilex* as a separate species from *A. draco*, even though future study is expected to evaluate their taxonomic status and geographic distribution more accurately. Voucher specimens were deposited in the Key Laboratory of Conservation and Application in Biodiversity of South China, Guangzhou University, Guangzhou (GU), and the Marine College of Shandong University at Weihai (SUS).

Examined specimens and collection localities are as follows: *Apodemus chevrieri* (*n*=11): Mt. Emei, Sichuan, GU MM3566 (male), 3593, 3594, 4478, 4480, 4484 (females), Wolong, Sichuan, SUS S1124, S1264, S1265 (males), S1107, S1236 (females); *Apodemus draco* (*n*=41): Mt. Emei, Sichuan, GU MM3545, 3563, 3564, 3568, 3569, 3570, 3585, 3586, 3596, 3599, 4479, 4483, 4485 (males), 3551, 3565, 3578, 3579, 3587, 3595, 4482 (females); Labahe, Tianquan, Ya’an, Sichuan, GU10073, 10076, 10077, 10094, 10107, 10128 (males), 10074, 10108, 10110 (females); Kangding, Sichuan, GU10137, 10139, 10148 (males), 10135, 10147 (females); Wolong, Sichuan, SUS S1140, S1257, S1266 (males), S1108, S1180, S1245, S1246 (females); *Apodemus ilex* (*n*=9): Ailaoshan, Xinping, Yunnan, SUS S570, S649, S661, S663, S667, S683 (males), S651, S662, S684 (females); *Apodemus latronum* (*n*=10): Kangding, Sichuan, GU10134, 10157 (males), 10136, 10140, 10145, 10151, 10153 (females), Wolong, Sichuan, SUS S1136, S1156 (males), S1134 (female).

Cytological preparations were made from tail and/or lung tissue culture cells using the standard air-drying method described by Harada & Yosida (1978). C-band staining was accomplished as per Sumner (1972) for selected species and specimens. Terminology for chromosomes followed Levan et al. (1964): i.e., metacentric, submetacentric, subtelocentric, and acrocentric. Diploid chromosome number (2*n*) and fundamental number of autosomal arms (FNa) were calculated.

## RESULTS

**Figure 1:**
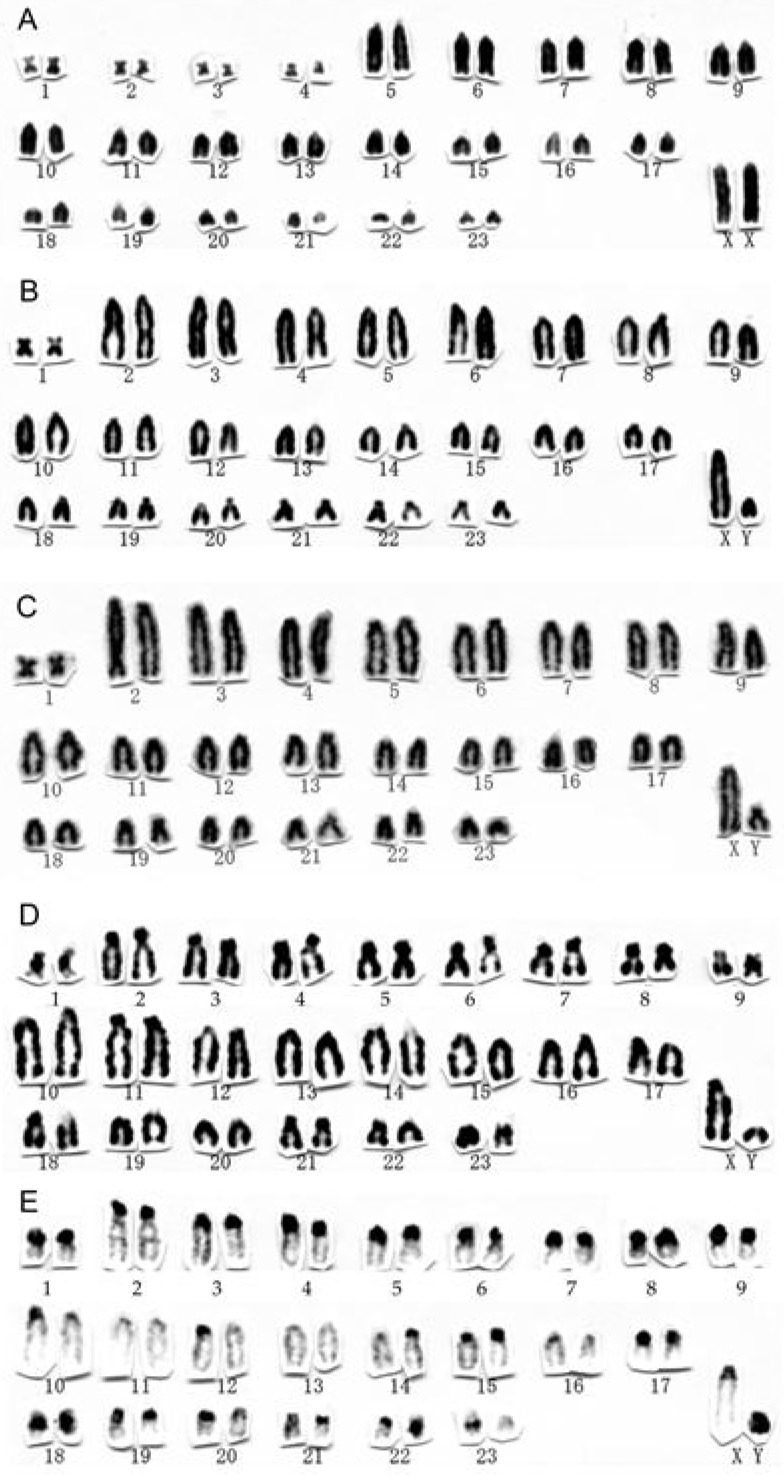
Karyotypes of *Apodemus* species from China

The karyotype of *Apodemus chevrieri* (Figure 1A) consisted of four small meta- or submetacentric pairs (nos. 1–4) and 19 large-to-small acrocentric pairs (nos. 5–23) in autosomes, large acrocentric X chromosome, and small acrocentric Y chromosome. The 2*n* and FNa values were 48 and 54, respectively.

Conventional karyotypes of *A. chevrieri* (A, GU MM3593), *A. draco* (B, M10077), *A. ilex* (C, SUS S649), and *A. latronum* (D, GU10134), as well as the C-band karyotype of *A. latronum* (E, GU10134).

The karyotype of *Apodemus draco* (Figure 1B) consisted of one small metacentric pair (no. 1) and large-to-small acrocentric pairs (nos. 2–23) in autosomes, large acrocentric X chromosome, and small acrocentric Y chromosome. The 2*n* and FNa values were 48 and 48, respectively.

The karyotype of *Apodemus ilex* (Figure 1C) consisted of one small metacentric pair (no. 1) and large-to-small acrocentric pairs (nos. 2–23) in autosomes, large acrocentric X chromosome, and small acrocentric Y chromosome. The 2*n* and FNa values were 48 and 48, respectively.

The karyotype of *Apodemus latronum* (Figure 1D) consisted of one small submetacentric (no. 1) and 22 large-to-small (nos. 2–23) acrocentric pairs in autosomes, large acrocentric X chromosome, and small acrocentric Y chromosome. In several acrocentric pairs, the centromeric region was well developed due to the constitutive heterochromatins, which were well stained following C-band staining (Figure 1E, nos. 2–9). As we could not find clear short arms for those pairs, we considered those pairs to be acrocentric. The 2*n* and FNa values were 48 and 48, respectively.

## DISCUSSION

We analyzed the karyotypes of four *Apodemus* species from China. Previous karyotypic data from this genus are summarized in Table 1, together with our results from this study.

**Table 1:** Karyotypes of field mice of the genus *Apodemus* examined in this study and reported in previous studies

Species	Locality	2*n*	FNa	M/SM	ST	A	X	Y	B	Reference
*A. chevrieri*	Sichuan, China	48	54	4	0	20	A	A	–	This study
*A. agrarius*	Shandong, China	48	54	4	0	19	A	A	–	Wang et al. (1993)
	Taiwan, China	48	56	5	0	18	A	A	–	Tsuchiya (1979)
	Korea	48	54	4	0	19	A	A	–	Kang & Koh (1976), Koh (1987, 1988, 1989),
										Matsubara et al. (2004)
	Primorye	48	52	3	0	20	A	A	–	Chernukha et al. (1986)
	Primorye	48	52–54	3–4	0	19–20	A	A	0–1	Kartavtseva & Pavlenko (2000)
	Amur	48	52	3	0	20	A	A	–	Kartavtseva & Pavlenko (2000)
	Khasan	48	54	4	0	19	A	A	–	Boeskorov et al. (1995)
	Khabarovsk	48	52–54	3–4	0	19–20	A	A	0–1	Chernukha et al. (1986), Kartavtseva (1994),
										Kartavtseva & Pavlenko (2000)
	Siberia	48	52–54	3–4	0	19–20	A	A	–	Boeskorov et al. (1995), Kartavtseva & Pavlenko (2000)
	Altai	48	52	3	0	20	A	A	–	Chernukha et al. (1986)
	Altai	48	54	4	0	19	A	A	–	Kartavtseva & Pavlenko (2000)
	Moskow oblast	48	52	3	0	20	A	A	–	Chernukha et al. (1986)
	Chechen-Ingush	48	52	3	0	20	A	A	–	Chernukha et al. (1986)
	Krasnodar	48	52	3	0	20	A	A	–	Chernukha et al. (1986)
	Ukraine	48	54	4	0	19	A	A	–	Kartavtseva & Pavlenko (2000)
	Moldova	48	52–54	3–4	0	19–20	A	A	–	Kartavtseva & Pavlenko (2000)
	Azerbaijan	48	54	4	0	19	A	A	–	Shbulatova et al. (1991)
	Czechoslovakia	48	54	4	0	19	A	A	–	Král (1970) (1972)
	Poland	48	54	4	0	19	A	A	–	Král (1970)
	Yugoslavia	48	54	4	0	19	A	A	–	Vujošević et al. (1984)
	Yugoslavia	48	52–54	3–4	0	19–20	A	A	–	Soldatović et al. (1969, 1975)
	Bulgaria	48	52–54	3–4	0	19–20	A	A	0–1	Chassovnikarova et al. (2009)
	Greece	48	54	4	0	19	A	A	–	Britton-Davidian et al. (1991)
	Turkey	48	54	4	0	19	A	A	–	Kefelioğlu et al. (2003)
	Turkey	48	56	5	0	18	A	A	–	Yiğit et al. (2000)
*A. draco*	Sichuan, China	48	48	1	0	22	A	A	–	This study
*A. ilex*	Yunnan, China	48	48	1	0	22	A	A	–	This study
	Yunnan, China	48	48	1	0	22	A	A	–	Chen et al. (1996) as “*A. peninsulae*”
*A. latronum*	Sichuan, China	48	48	1	0	22	A	A	–	This study
	Yunnan, China	48	66	8	2	13	A	?	–	Chen et al. (1996)
*A. semotus*	Taiwan, China	48	48	1	0	22	A	?	–	Matsubara et al. (2004)
*A. peninsulae*	Yunnan, China	48	46	0	0	23	A	A	–	Chen et al. (1996) as “*A. draco*”
	NE China	48	46	0	0	23	A	A	0–14	Wang et al. (2000)
	Korea	48	46	0	0	23	A	A	6–1	Koh (1986, 1988)
	Russia	48	46	0	0	23	A	A	0–6	Kartavtseva et al. (2000)
	Hokkaido, Japan	48	46	0	0	23	A	A	0–13	Hayata (1973)
*A. speciosus*	Japan	46–48	54	4–3	1	17–19	A	A	–	Tsuchiya (1974)
	Japan	46–48	54	5–4	0	17–19	A	A	–	Saitoh & Obara (1986)
*A. argenteus*	Japan	46	50	2	0	20	SM	A	0–1	Yoshida et al. (1975), Obara & Sasaki (1997)
*A. gurkha*	Nepal	48	50	2	0	21	A	?	–	Matsubara et al. (2004).
	Nepal	48	62–64	4–3	5	14–15	A	A	–	Gemmeke & Niethammer (1982)
*Sylvaemus* Group										
*A. sylvaticus*		48	46	0	0	23	A	A	–	Zima & Král (1984), Orlov et al. (1996),
										Kryštufek & Vohralík (2009)
*A. flavicollis*		48	46	0	0	23	A	A	1–3	Zima & Král (1984), Orlov et al. (1996),
										Kryštufek & Vohralík (2009)
*A. microps*		48	46	0	0	23	A	A	–	Zima & Král (1984), Reutter et al. (2001)
*A. alpicola*		48	46	0	0	23	A	A	–	Reutter et al. (2001)
*A. witherbyi*		48	46	0	0	23	A	A	–	Orlov et al. (1996), Kryštufek & Vohralík (2009)
*A. uralensis*		48	46	0	0	23	A	A	–	Orlov et al. (1996), Kryštufek & Vohralík (2009)
*A. ponticus*		48	46	0	0	23	A	A	–	Orlov et al. (1996)
*A. pallipes*		48	46	0	0	23	A	A	–	Gemmeke & Niethammer (1982)
*A. epimelas*		48	48–50	1–2	0	21–22	A	A	0–1	Belcheva et al. (1988), Zima & Král (1984)
*A. mystacinus*		48	50	2	0	21	A	A	–	Kryštufek & Vohralík (2009)

Diploid and sex chromosomes were classified into metacentric (M), submetacentric (SM), subtelocentric (ST), and acrocentric (A), and a "?" indicate the Y chromosome was too small to be confirmed. 2*n* and FNa, excluding the B chromosome. –: Not available.

The karyotype of *A. chevrieri* is reported here for the first time, and was characterized by four small metacentric pairs (2*n*=48, FNa=54). *Apodemus chevrieri* is restricted to southwestern China and based on mitochondrial and nuclear gene phylogenetic studies is thought to be a sister or in-group species of the widely distributed *A. agrarius* (Liu et al., 2004; Suzuki et al., 2003, 2008). Although the karyotype of *A. agrarius* is polymorphic and possesses 3–5 biarmed metacentric autosome pairs (2*n*=48, FNa=52–56, excluding the B chromosome; Boeskorov et al., 1995; Britton-Davidian et al., 1991; Chassovnikarova et al., 2009; Chernukha et al., 1986; Kang & Koh, 1976; Kartavtseva, 1994; Kartavtseva & Pavlenko, 2000; Kefelioğlu et al., 2003; Koh, 1987, 1988, 1989; Král, 1970, 1972; Matsubara et al., 2004; Shbulatova et al., 1991; Soldatović et al., 1969, 1975; Tsuchiya, 1979; Vujošević et al., 1984; Wang et al., 1993; Yiğit et al., 2000), the karyotype with four metacentric pairs (2*n*=48, FNa=54) is regarded as the standard karyotype for *A. agrarius* (see Kartavtseva & Pavlenko, 2000). Therefore, we suggest that there are no clear differences in the conventional karyotypes between *A. chevrieri* and *A. agrarius*; however, further study using differential staining of chromosome arms is expected to clarify any minor differences and rearrangement of chromosome arms between *A. chevrieri* and polymorphic *A. agrarius*, and thus help reevaluate their taxonomic status.

The karyotypes of *A. draco* and *A. ilex* are reported in this study for the first time as correct species identification, with both characterized by one small metacentric pair (2*n*=48, FNa=48), similar to that of *A. semotus* in Taiwan, China (Matsubara et al., 2004; Tsuchiya, 1979). While Chen et al. (1996) reported karyotypes of *A. draco* as 2*n*=48, FNa=46 and *A. peninsulae* as 2*n*=48, FNa=48 from Yunnan Province, China, these two karyotypes were possibly reported based on erroneous identification. We suggest that the former specimens collected from Kunming were *A. peninsulae*, whereas the latter specimens collected from Jianchuan were *A. ilex*. This interpretation of misidentification by Chen et al. (1996) would be congruent with the distribution of *A. draco* (currently *A. ilex*) in Kunming and Jianchuan and *A. peninsulae* in Kunming but not Jianchuan (Zhang, 1997); and that these two species have been considered superficially similar in morphologies and often misidentified before the careful taxonomic revision by Musser et al. (1996).

The karyotype of specimens of “*A. draco*” by Chen et al. (1996), and herewith interpreting to represent *A. peninsulae* showed no differences with the reported *A. peninsulae* karyotype and had only acrocentric chromosomes (2*n*=48, FNa=46; Hayata, 1973; Kartavtseva et al., 2000; Koh, 1986, 1988; Wang et al., 2000). The karyotype of the latter specimens correctly representing *A. ilex* was very similar to the karyotype for *A. ilex* from Yunnan, as well as *A. draco* from Sichuan in this study (2*n*=48, FNa=48) and *A. semotus* from Taiwan, China (2*n*=48, FNa=48; Matsubara et al., 2004; Tsuchiya, 1979) characterized by one small metacentric pair. Although the current study was limited to conventional karyotypes, we report here on the karyotypes of *A. draco* and *A. ilex* for the first time and provide updated information on the karyotype of *A. peninsulae*. These data are important for further study on species taxonomy and identification of the genus *Apodemus* in East Asia.

The karyotype of *A. latronum* was 2*n*=48 and FNa=48, with one small biarmed pair. This chromosome complement was similar to that of *A. draco*, *A. ilex*, and *A. semotus*, but the karyotype differed by having centromeric heterochromatin in many acrocentric pairs. Similar centromeric heterochromatin has been found in previous study on the karyotype of *A. latronum* from Yunnan Province (Chen et al., 1996). Chen et al. (1996) stated that the centromeric heterochromatin formed short arms and thus considered the *A. latronum* karyotype to be 2*n*=48, FNa=66. Although we did not analyze the G-band karyotype of *A. latronum*, based on the C-band karyotype we found no considerable differences between our *A. latronum* karyotype (2*n*=48, FNa=48) and that of Chen et al. (1996) (2*n*=48, FNa=66), despite different FNa values due to the interpretation of centromeric heterochromatin.

We studied the karyotypes of all *Apodemus* species in East Asia and provided a solid overview of chromosome evolution and species differentiation of the genus within East Asia. The chromosome rearrangements in East Asian *Apodemus* were congruent with the species divergence pattern proposed in previous molecular study (Suzuki et al., 2008). Suzuki et al. (2008) recognized four groups as the major DNA phylogenetic clades of the East Asian *Apodemus* subgeneric group: (1) *A. agrarius*–*A. chevrieri* (=*agrarius* species group), (2) *A. draco*–*A. ilex*–*A. semotus*–*A. latronum* (=*draco* species group), (3) *A. peninsulae*, and (4) *A. speciosus*. Suzuki et al. (2008) stated that these four groups radiated 6 Ma in response to global environmental changes among allopatric populations. Our present study clarified that these four DNA phylogenetic species groups were distinct, with different karyotypes: 2*n*=48, FNa=54 for the *agrarius* group (*A. agrarius*, *A. chevrieri*); 2*n*=48, FNa=48 for the *draco* group (*A. draco*, *A. ilex*, *A. semotus*, *A. latronum*); 2*n*=48, FNa=46 for *A. peninsulae*; and 2*n*=46/48, FNa=54 for *A. speciosus* (Tsuchiya, 1974; Saitoh & Obara, 1986. We suggest that these major chromosome rearrangements among clades played an important role in clade formation through post-mating reproductive isolation, in addition to allopatric distribution.

**Figure 2:**
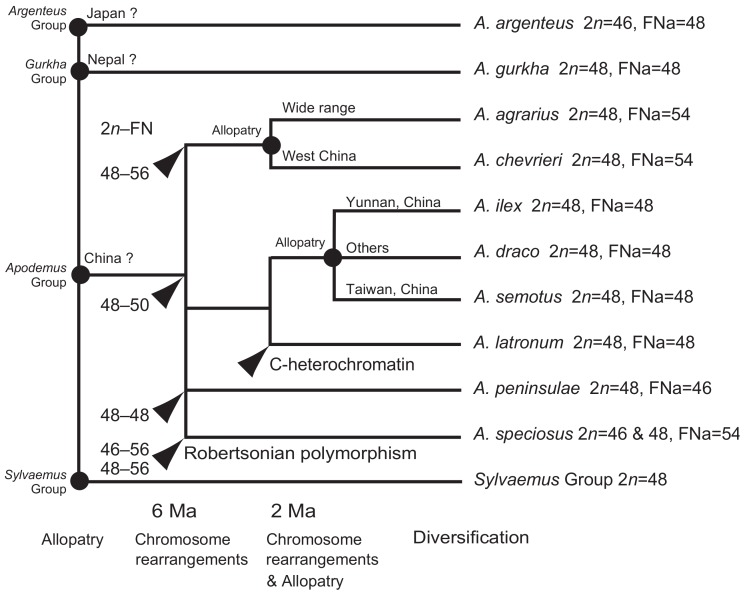
Hypothesized diversification process with allopatric distribution and chromosome changes in the genus *Apodemus* in East Asia

After the radiation into four groups, further speciation events are thought to have occurred within the *draco* and *agrarius* groups around 2 Ma (Suzuki et al., 2008). In the *draco* group, speciation likely occurred through allopatric speciation due to partitioning of the distribution range in developping geographic barriers, such as among *A. ilex* (Yunnan), *A. draco* (other areas in mainland China), and *A. semotus* (Taiwan, China), with minor chromosome rearrangements unlikely to have contributed to the speciation events of these three allopatric species (Figure 2). On the other hand, the current distribution range between *A. latronum* and *A. draco* and between *A. latronum* and *A. ilex* overlap (e.g., Musser et al., 1996). This suggests that *A. latronum*, which is distributed in the western provinces of Sichuan, Yunnan, Xizang, and Qinghai, as well as northern Myanmar (Musser & Carlenton, 2005), was not derived through allopatric speciation among the *draco* group. We propose that speciation of *A. latronum* from the *A. draco*-*A. ilex*-*A. semotus* clade may have occurred as sympatric speciation, where chromosome rearrangements contributed to form post-mating reproductive isolation at the cytological level. The increased centromeric heterochromatin found in *A. latronum* also influenced post-mating reproductive isolation from the *A. draco*-*A. ilex*-*A. semotus* clade, which lacked heterochromatin increase (Figure 2). On the other hand, *A. agrarius* and *A. chevrieri* in the *agrarius* group exhibit slight overlap in their current distribution ranges (Musser et al., 1996); and these two species may have undergone speciation by allopatric distribution, with subsequent expansion and overlap of their distribution ranges, as discussed by Suzuki et al. (2008). The speciation of *A. chevrieri* from *A. agrarius* is, therefore, suggested to have been accompanied by allopatric speciation events, and this evolutionary story may explain the lack of major karyotypic differences between the two species.

Phylogenetic relationships among species followed the molecular phylogeny of Suzuki et al. (2008). Some chromosome rearrangements referred to Matsubara et al. (2004). Arrowheads and closed circles indicate possible chromosome rearrangements and allopatric speciation, which resulting reproductive isolation. ?: Indicate the hypothetical origination of the clade/lineage.

In addition, extensive geographical divergences within the species have been reported for morphological and genetic traits in East Asian *Apodemus* species: e.g., *A. chevrieri* (Yue et al., 2012), *A. agrarius* (Sakka et al., 2010), *A. draco* (Fan et al., 2012; Kaneko, 2010, 2012, 2015; Sakka et al., 2010), *A. ilex* (Kaneko, 2010, 2012, 2015; Liu et al., 2012), *A. latronum* (Kaneko, 2010, 2012, 2015; Li & Liu, 2014; Sakka et al., 2010), *A. semotus* (Hsu et al., 2001), *A. peninsulae* (Kaneko, 2010, 2012, 2015; Sakka et al., 2010; Serizawa et al., 2002), *A. speciosus* (Kageyama et al., 2009; Shintaku et al., 2012; Shintaku & Motokawa, 2016; Suzuki et al., 2004; Tomozawa et al., 2014; Tomozawa & Suzuki, 2008), and *A. argenteus* (Suzuki et al., 2004). These complex patterns are thought to have formed through geographic isolation and genetic exchange (e.g., *A. speciosus* between Robertsonian chromosome races; Shintaku & Motokawa, 2016; Suzuki et al., 2004; Tomozawa & Suzuki, 2008) after the formation of each species. More comprehensive analyses using morphology, chromosomes, and DNA markers are expected to clarify the complex evolutionary history of the *Apodemus* genus in East Asia. The present study elucidated the evolutionary pattern of the *Apodemus* genus in East Asia with reference to the major chromosome rearrangements at the among-species level. Future study of major and minor chromosome rearrangements at the within-species level using various chromosome arm staining techniques is expected. The genus *Apodemus* may be considered a good wild animal model to understand the roles of reproductive isolation by allopatric distribution and chromosome rearrangement during speciation events.
